# Pathogen genomic surveillance and the AI revolution

**DOI:** 10.1128/jvi.01601-24

**Published:** 2025-01-29

**Authors:** Spyros Lytras, Kieran D. Lamb, Jumpei Ito, Joe Grove, Ke Yuan, Kei Sato, Joseph Hughes, David L. Robertson

**Affiliations:** 1 Division of Systems Virology, Department of Microbiology and Immunology, The Institute of Medical Science, The University of Tokyo, Tokyo, Japan; 2 MRC-University of Glasgow Centre for Virus Research 155698 , Glasgow, Scotland, United Kingdom; 3 School of Computing Science, University of Glasgow 315293 , Glasgow, Scotland, United Kingdom; 4 International Research Center for Infectious Diseases, The Institute of Medical Science, The University of Tokyo, Tokyo, Japan; 5 School of Cancer Sciences, University of Glasgow 3526 , Glasgow, Scotland, United Kingdom; 6 Cancer Research UK Scotland Institute 706312 , Glasgow, Scotland, United Kingdom; 7 Graduate School of Medicine, The University of Tokyo, Tokyo, Japan; 8 Graduate School of Frontier Sciences, The University of Tokyo 105219 , Kashiwa, Japan; 9 International Vaccine Design Center, The Institute of Medical Science, The University of Tokyo, Tokyo, Japan; 10 Collaboration Unit for Infection, Joint Research Center for Human Retrovirus Infection, Kumamoto University 624497 , Kumamoto, Japan; Indiana University Bloomington, Bloomington, Indiana, USA

**Keywords:** pLM, genomic surveillance, AI, language models, virus surveillance, public health

## Abstract

The unprecedented sequencing efforts during the COVID-19 pandemic paved the way for genomic surveillance to become a powerful tool for monitoring the evolution of circulating viruses. Herein, we discuss how a state-of-the-art artificial intelligence approach called protein language models (pLMs) can be used for effectively analyzing pathogen genomic data. We highlight examples of pLMs applied to predicting viral properties and evolution and lay out a framework for integrating pLMs into genomic surveillance pipelines.

## INTRODUCTION

Genomic pathogen sequencing during the COVID-19 pandemic highlighted the critical role of surveillance in understanding the persistence of SARS-CoV-2 in the human population despite high levels of protective immunity and the effect of the changing circulating variants on public health, advising policymakers and informing vaccine design. With nearly 20 million SARS-CoV-2 genomes currently available in the GISAID database, the sequencing effort since 2020 has surpassed those of other viruses, followed by less than half a million influenza virus genomes (GISAID) and less than 50,000 HIV-1 genomes (GenBank) all collected over several decades. The almost real-time resolution of circulating SARS-CoV-2 variants allowed scientists to readily detect and characterize genome changes corresponding to the virus’s functional and antigenic evolution in humans and anthroponosis events to other animals. This phenotypic change was apparent from the outset of the pandemic, with genomic surveillance revealing the first “functional” substitution, D614G (1, 2), and first significant antigenic substitution, N439K (3), both changes in the virus’s Spike entry glycoprotein. In addition to such point mutations creating variants with altered phenotypic properties, the global sequencing effort uncovered several mechanisms this new human virus used to maintain its circulation in the population: recurring indel mutations generating new variants, e.g., deletion H69/V70 (4); independent occurrence of the same amino acid substitutions at the same site in different variants (convergence) (5, 6); the emergence of variants of concern associated with multiple beneficial mutations through saltation-like evolution linked to chronic infections (7, 8); and bringing novel sets of mutations together in one variant (recombination) (9, 10), which can have a combinatory beneficial effect for the virus (positive epistasis) (11).

The scientific and public health communities’ response to the COVID-19 pandemic has been an unprecedented effort to track a novel virus’s evolution in near real time. However, SARS-CoV-2 surveillance was fragmentary with global disparities in coverage (12), and as the COVID-19 pandemic is now deemed over, sequencing has been downscaled. Given the general failure to learn the importance of sufficient levels of widespread sequencing to understand novel viruses, it is thus optimistic to expect that the same amount of effort will be put into understanding the next virus that spills over into humans. In fact, many viruses that have a high potential for impacting global health—but primarily affect resource-limited countries, such as the six priority virus pathogens causing human disease (MERS, Lassa, Nipah, Rift Valley Fever, Chikungunya, and Ebola) defined by the Coalition for Epidemic Preparedness Innovations—are largely under-surveilled. In addition to sequencing efforts, insights on pathogen evolution are further powered by time- and resource-consuming computational analyses, as well as validation with experimental approaches, such as *in vitro* deep mutational scanning (DMS) (13, 14). Advocating for more funding for pathogen genomic surveillance is undeniably the best approach forward; however, other priorities and resource limitations have made this approach disappointingly underwhelming so far (15). This leaves us with the need to develop enhanced computational approaches for answering the following questions: how do we monitor SARS-CoV-2 evolution and that of other endemic viruses when surveillance is diminished? How do we characterize the evolutionary landscape of neglected pathogens without knowing their underlying diversity? And can we predict which viruses have the highest zoonotic potential and prepare ourselves for an outbreak of a truly novel so-called “disease X” pathogen?

The field of artificial intelligence (AI) has recently experienced a boom in popularity and commercial applications, particularly in the form of large language models (LLMs). LLMs are state-of-the-art deep learning models trained on a large text corpus (set of words in the context of sentences) that identify fundamental properties of grammar and meaning (16). As such, the models can predict how changing a word can alter the meaning of a sentence given its understanding of these words in the specified context. In the same way, LLMs can be trained in any other corpus of contextualized characters, for example, amino acids in protein sequences, i.e., a protein language model (pLM) (17). Hie et al. (18) proposed that language models trained on protein sequences, instead of words, can be used to represent biological and biochemical properties of viruses. By introducing or “embedding” a new sequence into the model’s latent representation space, the potential effect of amino acid substitutions in a given peptide sequence can be predicted (19). Applied in the context of virus entry glycoproteins, the authors suggest that the changes in the “meaning” of a protein correspond to antigenic change, while changes in the “grammar” of the protein reflect changes in viral fitness. A number of pLMs have been developed recently (20), a popular example being Evolutionary Scale Modeling (ESM), trained, in the case of ESM-2, on over 60 million unique protein sequences covering all known biological diversity (21, 22). The key merit is that these models have a generalized and transferable “understanding” of proteins and, therefore, can be used to analyze completely novel sequences without the need for a multiple sequence alignment as required by, e.g., AlphaFold models (23). So far, the ESM family of models has proven revolutionary for quick and efficient predictions of proteins’ tertiary structure and protein design (22, 24, 25). But can the AI techniques be used for effective pathogen genomic surveillance?

Two of our recent studies have combined ESM-2 with the available SARS-CoV-2 genomic surveillance data to make inferences and predictions about the virus’s biological properties and evolution. First, on the premises of the protein “meaning” and “grammar” scores proposed by Hie et al. (18), Lamb and colleagues (26) use an *in silico* deep mutational scanning approach where each site in the Spike glycoprotein is iteratively changed to each possible amino acid residue. The model can then predict which sites are likely to accommodate substitutions and which changes are likely to be beneficial or deleterious for the virus, providing a high-level understanding of the effect substitutions may have on the protein function and structure. This information is engraved in protein evolution but may not be captured by high-throughput *in vitro* DMS methods, such as combinatorial antigenic changes and epistatic substitutions. Second, Ito and colleagues (27) adapt the ESM-2 architecture on three layers of information: (i) the reproductive number associated with each SARS-CoV-2 variant that has circulated in the past, (ii) antibody evasion *in vitro* DMS data, and (iii) Spike sequences representing the wider diversity of the *Coronaviridae*. This process allows the model to learn about the properties of variants that encode distinct Spike proteins and the effect that substitutions on these proteins have on evading immunity. Combining this knowledge on SARS-CoV-2 evolution with ESM-2’s understanding of protein language, the authors create a model that can accurately predict the reproductive number of past and future SARS-CoV-2 variants, simply based on the virus’s Spike protein sequence. Hence, models like ESM-2, which need little to no input on the circulating sequence diversity or the mechanisms behind virus evolution, can provide rapid valuable insights into pathogen biology.

Another innovative new method for viral escape forecasting has shown how using the known diversity of related viruses can largely improve the predictive power of such models (28, 29). Along the same lines, pLMs can be fine-tuned on specific groups of peptides to improve the model’s understanding of that group’s unique biological properties (30). We can imagine a starting model such as ESM-2, pretrained on all known proteins, that is subsequently fine-tuned on the proteome of a virus group with members affecting global health, or even all known virus receptor-binding proteins. Embedding a single protein sequence of a new virus genome into such a model would produce actionable predictions on viral fitness and antigenicity, as well as identify substitutions that may drive these functional changes. This process does not require an alignment and is essentially agnostic to the unsampled diversity of the circulating pathogen. Even though genomic surveillance is still necessary, only a fraction of the circulating diversity needs to be sampled for these models to provide predictive insights into the pathogen’s function and potential evolutionary trajectories. The current AI models’ usefulness does not extend to epidemiological insights, but sequencing is not required for this, with multiple cheaper and more widely available diagnostic tools existing for most viral pathogens.

**Fig 1:**
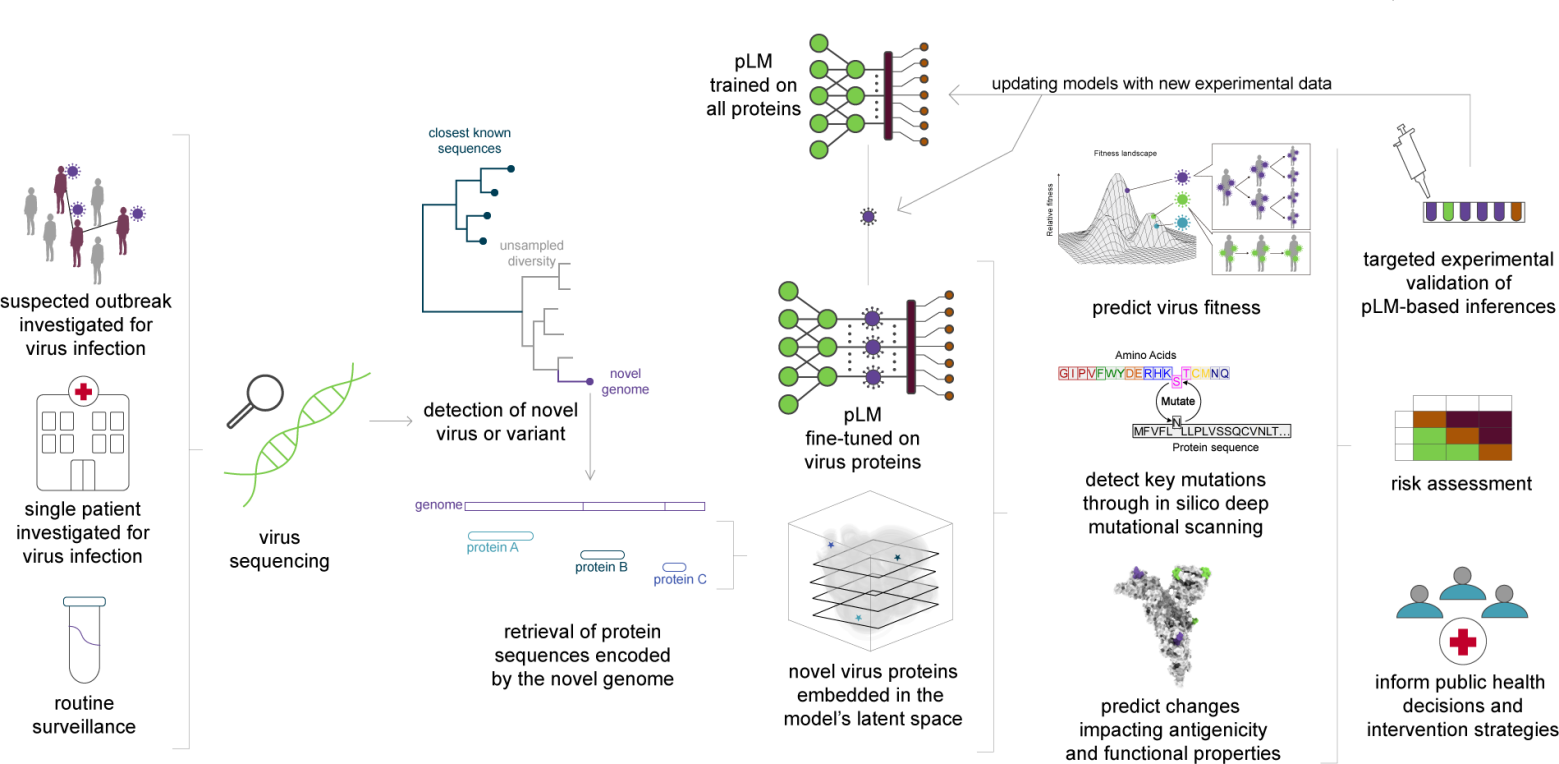
Overview of integrating pLMs to pathogen genomic surveillance pipelines to more effectively inform public health decisions on potential outbreaks.

It is clear that AI and protein language models hold great and mostly untapped potential for making genomic pathogen surveillance more effective, responsive, and less costly. The COVID-19 pandemic has ignited the development of extensive genomic surveillance infrastructure globally, which will likely be applied in future public health emergencies. Now, as part of ongoing preparedness efforts, is the right time to urge scientists developing the computational side of such infrastructure to consider implementing pLM methods in their pipelines (Fig. 1). Of course, improvements can be made to the methods themselves, for example, expanding and balancing the data sets these models are trained on (31). A better understanding of overall protein diversity—and specifically virus diversity—can largely aid the predictive ability of these models; thus, we note the paramount importance of animal virus discovery in pandemic preparedness. Not only for finding where viruses with zoonotic potential reside but also for improving the predictive models’ understanding of virus biology and for rational design of drug and vaccine interventions.

The inherent caveat of machine learning approaches, being restricted by the diversity of the data sets they are trained on, means that predictiveness can reach an “out-of-distribution” problem. That is, genuine inferences about a protein sequence can only be made if it falls within the distribution of sequence contexts the model was trained on. Metagenomic virus discovery and high-throughput reanalysis of existing sequencing read data sets can largely aid in diversifying training data beyond the formally annotated viral protein diversity and expanding the models’ context distribution (32–34). In addition to diversity in the sequence training data set, the same caveat applies to functional data used for model training. Hence, aside from continuous virus surveillance and discovery, experimental molecular virology and phenotypic virus characterization—potentially driven by initial AI model predictions—should be regularly performed, and the resulting data used for training models (Fig. 1). The need for updating training data sets should naturally come hand-in-hand with frequently updating the models themselves. Even though training a pLM from scratch can be very computationally intensive, updating existing pLMs with specialized tasks on new data is substantially easier, making this technology extremely fitting for dynamically updating data sets (27).

In the presence of comprehensive epidemiological, sequencing, and phenotypic data for a virus, combinatorial predictive models can be developed that perform well for variant prediction (35). However, this breadth of data is rarely available. Despite the “out-of-distribution” problem described above, AI models do have the extrapolative capacity to make accurate predictions in the absence of comprehensive data. The future of pathogen preparedness should focus on efficiency and effectiveness in translating data to successful public health policies and decisions. Integrating AI-driven methods into our routine preparedness pipelines will pave the way for making fast and informative risk assessments about novel pathogens. This approach reduces the need for comprehensive sequencing or immediate experimental validation, which can become targeted and frequently feedback to the models (Fig. 1).

When discussing the need for data to train AI models, we should also highlight the importance of effectively acknowledging data contributors. Acknowledging sequence submitters has been an important discussion throughout the COVID-19 pandemic where global availability of SARS-CoV-2 sequences was critical for public health decisions (36). For effective and equitable use of the AI-based technologies discussed here, we believe that all published models should have full transparency of their training data sets, including acknowledging all parties that led to the data collection. Notably, much of the sampling and sequencing takes place in resource-limited areas. Acknowledging these efforts is essential, but it must be accompanied by guarantees that data submitters can also benefit from the models trained on their contributions. Simply making model weights and code available is insufficient. In many regions, local computing resources are prohibitively expensive, while technical expertise is lacking, and necessary infrastructure may be unavailable. To address this, investment in AI development should include technical training for data contributors and establishing models available through cloud computing where only internet access is required for using the models instead of costly local infrastructure. This should apply to academia, but also—perhaps even more importantly—to industry since most “foundational” models to date have been developed by tech companies that hold the necessary resources and capital to support such projects. In these settings, ethics committees should be in place to enforce equitable AI model development. Ensuring that data providers are acknowledged and have full access to the resulting models is the only way to sustain continuous updating and, subsequently, the predictive performance of these models.

So far, genomic surveillance has complemented public policy after a viral outbreak has already occurred. The generalizable nature of pLMs has the potential to help us create transformative tools where a single genome sequence allows us to predict and respond to spillovers, understand variant effects (26), predict functional interactions (37), and what interventions are required before widespread transmission takes off in the human population, enabling truly actionable preparedness strategies. The compute capacity required to build such models will require access to intensive GPU resources and expertise not usually available to academic biologists. Interdisciplinary partnerships, know-how, and training will need to be in place in preparation for the next virus outbreak. Without a doubt, viruses will continue to spill over and cause outbreaks that we can prevent if we are equipped with the right tools; the time for action is now.
